# Effects of nanoscale zero-valent iron loaded biochar on the fate of phenanthrene in soil-radish (*Raphanus sativus* L*. var.radculus pers*) system

**DOI:** 10.1016/j.eehl.2025.100134

**Published:** 2025-01-22

**Authors:** Lianzhou Shen, Yue Cai, Juan Gao

**Affiliations:** a Institute of Soil Science, Chinese Academy of Sciences, Nanjing 211135, China; b University of Chinese Academy Sciences, Nanjing College, Nanjing 211135, China; c State Environmental Protection Key Laboratory of Environmental Health Risk Assessment, South China Institute of Environmental Science, Ministry of Ecology and Environment, Guangzhou 510655, China

**Keywords:** Bioaccumulation, Environmental effects, Microbial community, Nanomaterial, Oxidative stress

## Abstract

Nanoscale zero-valent iron loaded on biochar (nZVI@BC) has been proven to be effective in activating persulfate to remediate soil organic pollutants. However, studies on subsequent plant growth and microbial community changes in remediated soil remain limited. In this study, nZVI@BC, nZVI, and nanoscale biochar (nBC) were ball-mill produced and applied as amendments in pot experiments with PAH-contaminated soil to investigate their impacts on soil-crop (radish, *Raphanus sativus* L.) systems, and the widely distributed phenanthrene (Phe) was selected as model pollutant. The results indicate that nZVI@BC could induce more (75%) Phe accumulation in radish compared to the control treatment, but did not result in significant differences in plant biomass or enzyme activity. In Phe non-contaminated treatments, the Fe content of radish shoots increased from 86.87 ± 5.61 mg/kg DW without material application to 125.20 ± 11.93 mg/kg DW with nZVI@BC, while no significant differences were observed in roots. nZVI@BC and nBC increased the non-desorbed fraction of PAHs with low bio-availability by 13.6% and 10.2%, respectively, after 45 days compared to the control treatment. Illumina MiSeq sequencing revealed that nZVI@BC did not adversely affect the richness and diversity of soil microbial communities. Instead, it promoted the enrichment of bacteria related to the degradation of organic pollutants, such as *Lysobacter* and *Spingomonas*. The findings suggest that nZVI@BC after chemical oxidation remediation might be harmful to subsequent plants and ecosystems but much better than nZVI alone. The amount of nZVI@BC should be accurately calculated before chemical oxidation remediation.

## Highlights

• By promoting the conversion of soil phenanthrene to non-extractable states, nZVI@BC reduces its bioavailability, despite increasing the total amount present.
• While having no significant effect on radish seed germination or growth alone, nZVI@BC combined with Phe significantly shortens root length.
• The increase in root iron and manganese content caused by nZVI is effectively mitigated by nZVI@BC.
• The enrichment of Phe-degrading bacteria, such as *Sphingomonas* and *Lysobacter*, is promoted by nZVI@BC.

## Graphical abstract

**Figure:**
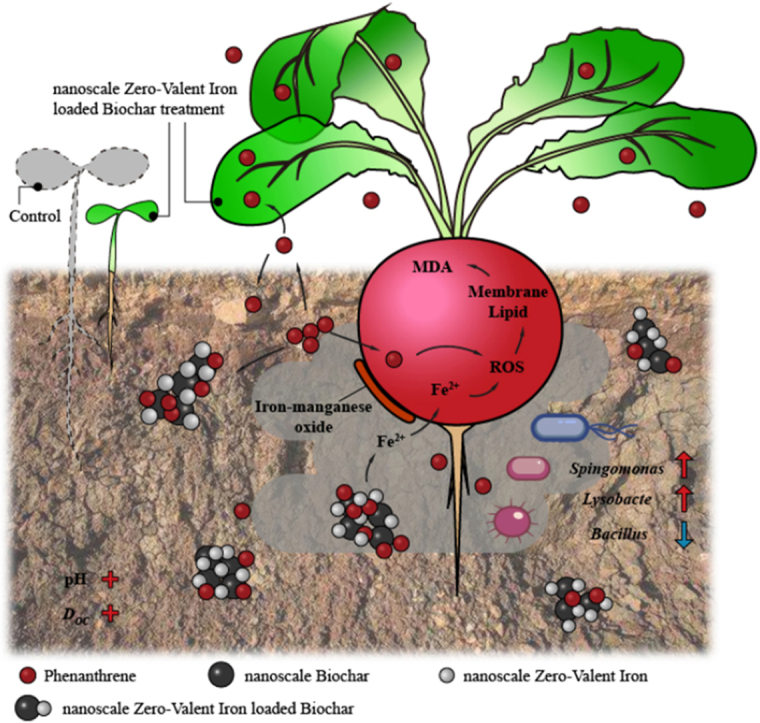

## 1 Introduction

Polycyclic aromatic hydrocarbons (PAHs), a major group of persistent organic pollutants, are of significant concern due to their teratogenic, mutagenic, and carcinogenic properties [1,2]. These substances in soil have the potential to be sequestered and accumulated by plants, thus posing a significant risk to human health through food chains [3]. PAHs have extensively accumulated in topsoil in China, with concentrations ranging from undetectable levels to 0.261 μg/kg [4]. The concentration of PAHs in industrial contaminated fields could be as high as 5872 μg/kg [5].

Chemical oxidation processes based on persulfate have received increasing interest in PAH-contaminated soil remediation in recent years due to their high efficiency [6]. Zero-valent iron (ZVI) materials, including nanoscale ZVI (nZVI), are considered as common and efficient activators of this technology [7]. However, subsequent biological toxicity and defects of nZVI, including a high aggregation tendency, easy oxidation, and high cost, bring many challenges to the widespread adoption of such technology [8]. To handle this problem, biochar (BC) was proposed as a viable supportive material for nZVI. BC is a valuable soil amendment that could increase the efficiency and stability of iron-based catalysts [9]. Based on this premise, biochar-based nano zero-valent iron (nZVI@BC) was produced [10] and demonstrated significant advantages in both controlled and field settings. On the laboratory scale, Yan et al. [11] enhanced 75.6% degradation efficiency of trichloroethylene by adding biochar and nZVI to enhance persulfate activation. In a field remediation study, Zeng et al. [12] reported a pilot chemical oxidation study for *in-situ* soil remediation with nZVI@BC and persulfate. The degradation efficiency of the target pollution, 2-ethylnitrobenzene, exceeded 99%. However, while exploring a new type of remediation material, its environmental friendliness should also be assessed. It has been demonstrated that nZVI@BC was significantly efficient in activating persulfate to produce reactive radicals during soil remediation. However, the high reactivity and biotoxicity of nZVI [13] and the fixation effect of biochar to Σ16PAHs in soil [14] have been recently reported, which indicated potential health hazards of nZVI@BC. Meanwhile, recent research has primarily focused on balancing the ecological benefits and efficiency of Fe-based material-loaded biochar composites, while often neglecting the mobility of nanoscale materials that can be transported to water through runoff. Therefore, it is essential to conduct a more comprehensive analysis of the complex material and investigate the dynamics between this material and a variety of ecosystem components in practical scenarios, which can help us thoroughly understand the effects and optimize its application.

Phenanthrene (Phe), commonly detected in contaminated fields [15], was selected as the model compound of PAHs in this study, and radish (*Raphanus sativus* L. *var. radculus pers*) was selected to represent vegetables growing in the remediated field. This study aimed to (1) investigate the influence of nZVI@BC on the bioavailability of Phe in soils for radish; (2) examine radish responses under the coexistence of Phe and nZVI@BC; (3) analyze the effects of nZVI@BC amendment on soil microbial communities. The study would be useful in thoroughly understanding the fate of nZVI@BC in soil and its ecological effects on the soil-plant system.

## 2 Materials and methods

### 2.1 Chemicals

Phe and pyrene (Pyr) were purchased from Shanghai Aladdin Bio-Chem Technology Co., Ltd. (Shanghai, China). Acetone (AR), dichloromethane (DCM, GR), and n-hexane (GR) were obtained from Merck & Co., Inc. (Darmstadt, Germany). Sulphuric acid (H_2_SO_4_, AR) and hydrogen peroxide (H_2_O_2_, AR) were obtained from Nanjing Chemical Reagent Co., Ltd. (Nanjing, China). Total protein quantification test kits and Malondialdehyde (MDA) test kits were obtained from Nanjing Jiancheng Bioengineering Research Institute (Nanjing, China). Chromatography silica gel (200–300 mesh) was obtained from Qingdao Ocean Chemical Co., Ltd. (Qingdao, China). Radish (*R. sativus* L. *var. radculus pers*) seeds were obtained from Jinshengda Seed Co., Ltd. (Nanjing, China). Zero-valent iron was purchased from Yalv Aluminum Material Co., Ltd. (Gongyi, China). Rice straw biochar was purchased from Xingnuo Environmental Protection Materials Co., Ltd. (Zhengzhou, China).

### 2.2 Soil sampling

Soil samples were collected from the top layer (0–20 cm) of a field located in Hengxi town, Nanjing, Jiangsu Province, China (118.7834612°E, 31.7259186°N). The soil sample was air-dried and sieved (2 mm, 10 mesh) after the removal of stones and plant roots. The soil pH was determined at a water-to-soil ratio of 1:2.5 (w:v) using a pH meter (PHS-3C, Leici, China). The dissolved organic carbon (DOC) was determined using a total organic carbon analyzer (Vario TOC Select, Elementar, Germany). The Beckman Coulter LS13320 laser particle size analyzer (USA) was employed to analyze the composition of soil particles.

Therefore, the mechanical composition of the experimental soil, having 72.1% silt, 0.2 g/kg of SOM, and a pH of 6.85, was consistent with the physical characteristics of typical yellow-brown soil. However, the concentration of Phe in the original soil was below the detection limit.

Each 2 kg soil sample was spiked with 500 mL of a 0.4% acetone solution of Phe, thoroughly mixed, and air-dried to prepare the primary Phe-contaminated soil. This soil was then gradually diluted with clean soil to achieve the desired concentration. After 14-d aging, the concentration of Phe was measured to be 9.16 ± 0.37 mg/kg. Based on previous studies [16], radishes can sustain normal growth and avoid death at PAH concentrations of 10 mg/kg while also showing physiological differences as compared to those under non-polluted conditions, such as variations in biomass and enzyme activity. Therefore, this selected concentration was helpful in statistical analysis in later evaluation.

### 2.3 Material preparation and characterization

Following a previous study [12], nZVI@BC, nZVI, and nanoscale biochar (nBC) were prepared using a Planet-type ball mill (XQM-100L, Tianchuang Powder Technology, Changsha, China). Detailed procedure is presented in Text S1. The particle sizes were determined with a dynamic light scattering instrument (DLS, Litesizer 500, Anton Paar, Austria). The results revealed that 44.1% of the nZVI@BC particle sizes were within the nanomaterial scale, ranging from 1 to 100 nm (Fig. S2). The content of Fe in the composite was determined using an o-phenanthroline colorimetric method [17] following digestion treatment.

To examine the impact of soil aging on the composites, three bags (8 cm × 6 cm each) were filled with corn fiber and equal quantities of nZVI@BC particles, which were then buried in the soil of pot experiments. These materials in bags were taken out after 45 d aging, freeze-dried, and crushed into powder. The composition and surface elements of nZVI@BC particles were analyzed using X-ray photoelectron spectroscopy (XPS, Escalab Xi+, ThermoFischer). The spectral deconvolution method was then used to identify the changes in surface functional groups using the XPS peak (Version 4.1, Raymund W.M. Kwok, Chinese University of Hong Kong).

### 2.4 Plant pot experiments

The study comprised sixteen treatment groups, each assigned to one of two primary soil conditions: contaminated (Cont) or non-contaminated (NCont). The soil conditions were further classified based on the presence or absence of plants (P or NP) and the incorporation of a variety of substances: a control group with no additional materials (C), a group added with nZVI (Z), a group added with nBC (B), and a group containing nZVI@BC (ZB). Details can be found in Table S1 and Fig. S1.

Glass Petri dishes were employed to sow 12 seeds per dish in the seedling experiments, containing either 71.4 g of amended soil or 70 g of unamended soil. Another seed batch was sown in plastic pallets (16 cm × 14 cm × 5 cm) filled with quartz sands. Ten seedlings were transferred to Petri dishes with identical conditions (1 cm of roots) and cultivated for an additional five days before being harvested. After that, the lengths of germs and radicles were measured.

In pot experiments, seeds were germinated in porcelain pots (7.5 cm × 5 cm × 9 cm) containing either 200 g of soil without amendments or 204 g of soil added with 2% amendments. The cotyledon height similarity was used to select one seedling per container five days after germination. The seedlings were subsequently developed in a greenhouse that was maintained at a temperature range of 15–25 °C. The positions of the pots were randomized every three days, and the soil moisture was maintained at 60% of its maximum capacity. Plants and soil were harvested at 40 d. The plants were washed with deionized water, and then shoots and roots were separated and weighed using an electronic balance (BSA323S-CW, Sartorius Scientific Instruments Co., Ltd., China). Parts of fresh plant samples were preserved at −80 °C before physiological and biochemical analyses; the remaining parts were freeze-dried and ground with liquid nitrogen for the following analysis.

### 2.5 Plant physiological characterization

The antioxidant activity in plants was assessed by measuring the total antioxidant capacity (TAC) and MDA concentrations. Samples were pre-treated separately with Ferric ion-reducing antioxidant power (FRAP) method [18] and MDA kits (Nanjing Jiancheng Bioengineering Research Institute, China) and analyzed at 562 and 532 nm with a microplate reader (Sunrise RC TS TC, Tecan, Austria). The inductively coupled plasma-atomic emission spectrometer (ICP-AES, Avio 200, Waltham PerkinElmer, USA) was employed to analyze the Fe and Mn contents of radishes following digestion. Detailed methods are provided in Text S2.

### 2.6 PAHs extraction and analysis

The extraction of plant Phe followed these steps: 0.05 g plant powder was deposited in a glass centrifuge tube, in which 0.5 mL Pyr acetone solution (2.5 mg/L) was preadded as an internal standard, then a mixture of 6 mL of an n-hexane and dichloromethane mixture (1:1, v:v) was added for extraction. For the extraction of Phe in soil, a 0.2 g soil sample was weighed and extracted with dichloromethane to ascertain the total Phe content of the soil (details in Text S3).

It was recommended [19] to extract different fractions of Phe in soil (Text S3), which could be used to indicate the levels of Phe bioavailability and toxicity. Gas chromatography-mass spectrometry (GC–MS, QP 2010 Plus, Shimadzu) was employed to analyze the extraction solutions, and each extraction experiment was conducted in triplicates. The detailed explanations of instrument parameters are presented in Text S4. The total recoveries of Phe in plant and soil samples ranged from 83.7% to 122% and 90.3% to 101.4%, respectively.

### 2.7 DNA extraction and Illumina MiSeq sequencing

DNA extraction and Illumina MiSeq sequencing were performed by Personalbio Biotechnology Co., Ltd. (Shanghai, China) following standard protocols. Quantitative Insights Into Microbial Ecology (QIIME, 2019.4) was primarily employed to conduct the analyses of sequence data and microbiome bioinformatics [20]. Complete experimental protocols are provided in Text S5. LEfSe and alpha- and beta-diversity were analyzed to identify variations in dominant genera and changes in community composition.

### 2.8 Quality control and statistical analysis

The data for each soil and plant indicator in this research were expressed using mean ± standard deviation. The data were subsequently processed and graphed by Origin 2017 (Origin Lab, USA). SPSS 26.0 (IBM, USA) was used to perform a one-way analysis of variance employing Duncan's multiple range tests to evaluate the significance of differences between interventions; a criterion of *p* < 0.05 was applied to determine significance.

## 3 Results and discussion

### 3.1 Characteristics of fresh and aged nZVI@BC

**Fig. 1:**
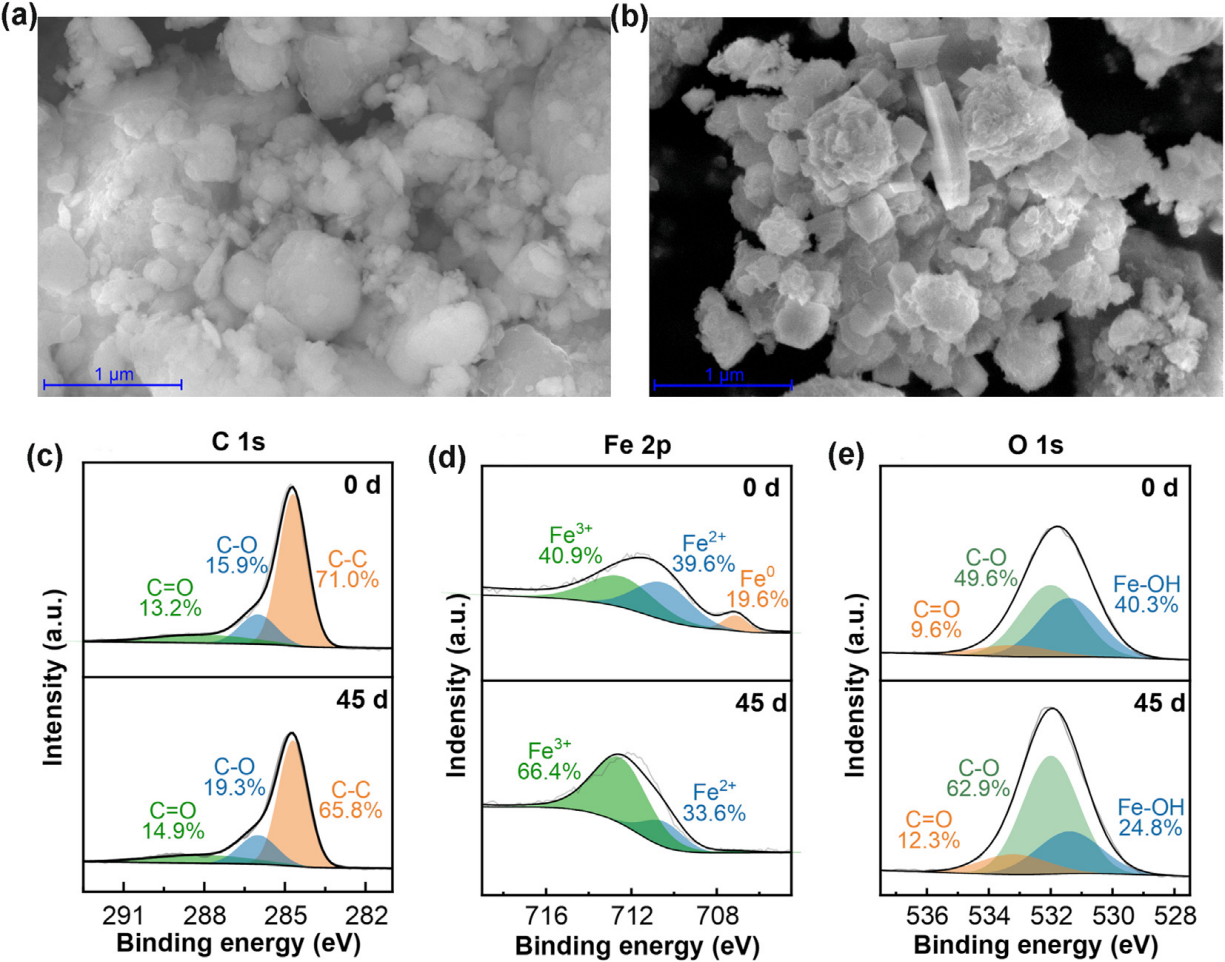
SEM images of fresh and used nZVI@BC (a, b) and XPS spectra of overall C 1s (c), Fe 2p (d), and O 1s (e) for fresh and used nZVI@BC.

The morphology of freshly synthesized nZVI@BC and aged particles after 45 d in soil is illustrated in Fig. 1a and b, respectively. The aged nZVI@BC showed a considerably higher quantity of small grain particles compared to fresh samples. The XPS and XRD examination revealed changes in the elemental composition (Fig. 1c–e and Fig. S3). The characteristic peaks of Fe 2p appeared at the binding energy of 706.6, 710.5, and 712.5 eV and were attributed to Fe^0^, Fe^2+^, and Fe^3+^, respectively [21,22]. The peak of Fe^0^, which was initially observed in fresh nZVI@BC, disappeared by 19.6% after 45-d aging. Conversely, the proportion of Fe^3+^ increased from 49.9% to 66.4% (Fig. 1c). The peaks of O 1s at the binding energy of 531.4, 532.0, and 533.2 eV indicated the presence of Fe–OH, C–O, and C=O bonds, respectively [23,24]. After aging, the proportion of O in the C–O peak increased from 49.6% to 62.9% (Fig. 1d), suggesting an increased formation of C–O bonds. The analysis of the C 1s spectrum revealed a slight variation in the proportions of C–C (284.7 eV), C–O (286.0 eV), and C=O (288.0 eV) peaks after pot experiments [23,24]. The proportions of C–C containing functional groups decreased from 71.0% to 65.8%, while the percentages of C=O and C–O groups increased in accordance with XPS findings in O 1s.

Li et al. [25] and Hui et al. [26] have previously investigated the aging of nZVI. It was postulated that nZVI underwent chemical reactions (Eqs. (1), (2), (3), (4)) in soil, resulting in the formation of magnetite, lepidocrocite, and goethite. The observed changes in the proportion of Fe^3+^ confirmed the occurrence of oxidation processes in soil. Furthermore, a decrease in C–C compositional percentages (from 71.0% to 65.8%) indicated the potential bioavailability of biochar in soil. The aforementioned behaviors demonstrate that the composites nZVI@BC could influence soil iron contents, thereby affecting soil pH, plant physiology, and Phe accumulation. The morphological characteristics of the nZVI@BC composite remained relatively stable in soil despite these chemical interactions, although there was a possible loss of small particles due to the process of aggregation. The observed reactivity suggests that nZVI@BC particles have the potential to continually alter soil properties, thereby possibly affecting plant growth and microbial communities in soil [27].

Table S2 shows the incorporation of three different materials increased soil pH ranging from 0.08 to 0.23 compared with the control group, which might be associated with the fact that nZVI could react with soil pore water and oxygen, leading to the formation of OH^−^ ions (Eqs. (3), (4)) [28,29], and nBC influenced soil pH by consuming protons through the decarboxylation of organic anions [30]. The addition of nZVI@BC and nBC increased soil DOC by 2.43%–12.58% in Cont-NP-ZB and by 15.69%–48.46% in Cont-NP-B groups, respectively.

(1) $${\text{Fe}}^{0}+2H_{2}O={\text{Fe}}^{2+}+H^{+}+2{\text{OH}}^{-}$$

(2) $$6{\text{Fe}}^{2+}+O_{2}+6H_{2}O=2{\text{Fe}}_{3}O_{4}+12H^{+}$$

(3) $$4{\text{Fe}}^{0}+3O_{2}+2H_{2}O=4\gamma -\text{FeOOH}$$

(4) $$4{\text{Fe}}_{3}O_{4}+O_{2}+6H_{2}O=12\gamma -\text{FeOOH}$$

### 3.2 Effects of nZVI@BC on radish growth

**Fig. 2:**
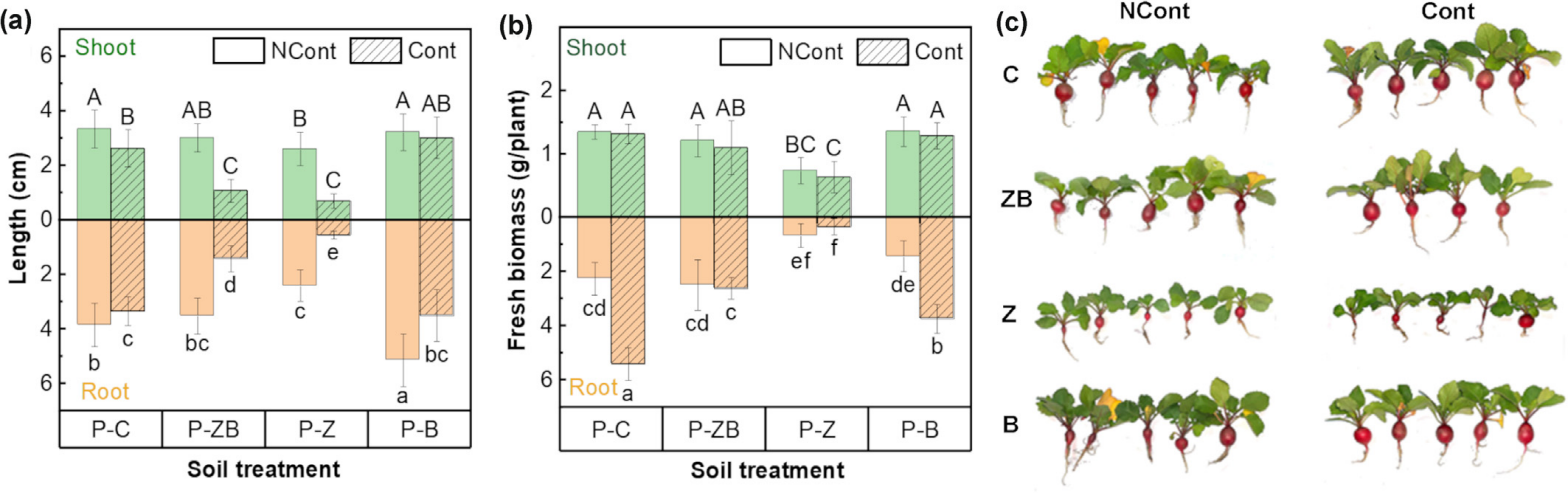
Seedling length (a), plant fresh biomass (b), and photographs (c) of radish in eight treatments after 45 d. Cont, contaminated soil; NCont: non-contaminated soil; P, plants; NP, non-plants; C, control; Z, nZVI treatment; B, nBC treatment; ZB, nZVI@BC treatment. Different uppercase and lowercase letters respectively represent significant differences in shoots and roots (*p* < 0.05).

The potential effects of various soil amendments on radish growth are illustrated in Fig. 2a and b. The root length in NCont-P-B treatment reached 5.12 ± 0.96 cm, which was significantly longer than that in the NCont-P-C treatment at 3.84 ± 0.79 cm (*p* < 0.05). Conversely, root elongation decreased to 2.40 ± 0.58 cm and 3.50 ± 0.66 cm, respectively, with the addition of nZVI and nZVI@BC. These findings suggest that nZVI had an adverse effect on the growth of radish, while nBC seemed to reduce the detrimental effects of nZVI. Radish seedlings grown in Phe-contaminated soils demonstrated poor growth (3.33 ± 0.53 cm) (Cont-P-C), and root elongation was merely 0.54 ± 0.14 cm with the addition of nZVI (Cont-P-Z). In Cont-P-Z, only 66.7% of seeds germinated, which was significantly lower than in Cont-P-C.

The above-ground biomass remained unaffected with the addition of nBC and nZVI@BC after 45-d cultivation; however, it was seriously affected by nZVI amendment (Fig. 2b). The average root biomass in Phe-contaminated soil was 5.39 ± 0.60 g, which was 2.40 times higher than that in uncontaminated soil (2.25 ± 0.61 g), indicating that low pollution levels could promote plant growth [31]. The root biomass of Cont-P-ZB was comparable to that of NCont-P-C but was substantially higher than that of Cont-P-Z (Fig. 2b and c and Fig. S4). This indicated that the composite nZVI@BC had no discernible effect on the growth of plants, while the nZVI component within nZVI@BC was identified as the main factor responsible for its toxicity.

### 3.3 Effect of nZVI@BC on Phe accumulation

**Fig. 3:**
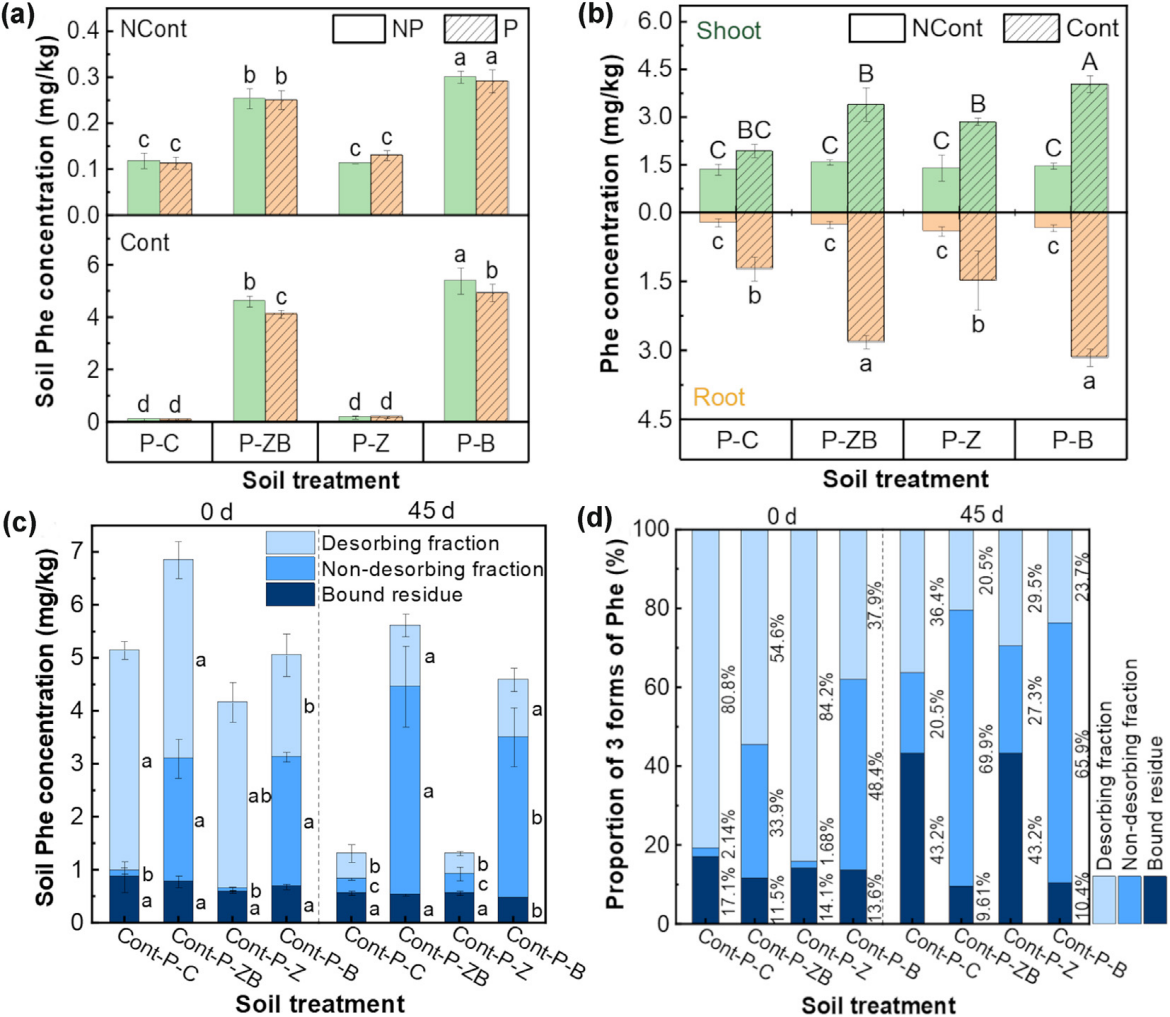
Soil Phe concentration in different treatments (a), plant Phe concentration in different treatments (b), and the concentration and proportion of 3 soil Phe fractions in different treatments (c, d). Different uppercase and lowercase letters respectively represent significant differences (*p* < 0.05).

Following a 45-d cultivation, total concentrations of Phe in soil were 4.63 ± 0.21 mg/kg for the Cont-P-ZB treatment and 5.40 ± 0.50 mg/kg for Cont-P-B treatment, which were 34.6 and 40.6 times higher than in Cont-P-C soil. Cont-P-Z treatment significantly decreased soil Phe concentration to only 0.20 ± 0.07 mg/kg (Fig. 3a). The accumulated Phe levels in both roots and above-ground parts were similar in all amendment treatments, and shoot levels were consistently higher than roots, indicating that foliar absorption from the atmosphere was a substantial Phe source (Fig. 3b). It has long been widely recognized that plants are important sinks of soil PAHs [32,33]. However, the NCont-P-C soil Phe concentrations were found to be 0.12 ± 0.02 mg/kg, because Phe evaporated from the contaminated soils in other treatment groups located within the same greenhouse [34]. Furthermore, soil Phe levels were 0.25 ± 0.02 and 0.30 ± 0.01 mg/kg in the NCont-P-ZB and NCont-P-B treatments, respectively, due to the enhanced adsorption of Phe in the atmosphere by added nZVI@BC and nBC in the soil.

The incorporation of nZVI@BC and nBC particles into Phe-polluted soil (Fig. 3c–d) resulted in 13.6% and 10.2% greater non-desorbed fractions of Phe after 45-d cultivation as compared with Cont-P-C, respectively. Moreover, the desorbed fraction of Phe was considerably decreased by these amendments, indicating that nZVI@BC and nBC both effectively reduced the bioavailability of Phe in soil, and nBC was identified as the primary contributor to this effect. The aforementioned results suggested that biochar played a critical role in soil by immobilizing Phe and then increasing the accumulation of Phe in radish. Soil Phe levels in Cont-P-ZB and Cont-P-B treatments were significantly higher than that of Cont-P-C, and the proportions of non-desorbed Phe were also considerably higher compared with the treatment without amendment and with nZVI. The degradation of PAHs was accelerated by the addition of nZVI [35], because nZVI produced Fe^2+^ and ·OH radicals upon reacting with O_2_. The addition of nZVI@BC to soil induced a conversion of Phe from the desorbed to the non-desorbed fraction. This conversion was facilitated by BC components, which participated in adsorption, chemical bonding, and physical encapsulation mechanisms [36], such as pi–pi interactions and pore-filling effects [37]. The mobility and bioavailability of PAHs were significantly reduced, suggesting biochar-derived substances significantly influence the fate of Phe. In addition, PAHs could also be taken up by plants via nanoparticle carriers [34].

### 3.4 Effects of nZVI@BC on plant oxidative stress

**Fig. 4:**
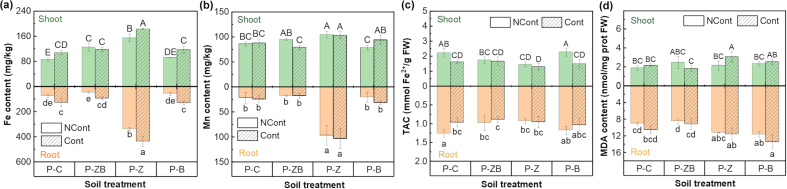
Fe, Mn contents (a, b), Total antioxidant capacity (TAC) (c), and malonaldehyde (MDA) content (d) of plants in different treatments. Prot, protein. Different uppercase and lowercase letters respectively represent significant differences in shoots and roots (*p* < 0.05).

Fig. 4a and b depict the Fe and Mn concentrations of radishes subjected to various interventions. With the addition of nZVI@BC and nZVI to the Phe-polluted treatments, the Fe content of radish shoots increased to 118.67 ± 7.18 and 183.60 ± 1.41 mg/kg DW, respectively. In contrast, incorporating nBC did not lead to any further increase in Fe content. The nZVI treatment effectively increased the Fe content in radish roots by a factor of 4.60 in NCont-P-Z compared to the NCont-P-C. Furthermore, the addition of nZVI significantly increased the Mn content in both radish roots and shoots (Fig. 4b). The observed co-increase could potentially be attributed to the continual exposure of roots to an iron-rich environment, leading to the formation of iron-manganese oxides on the root surface [38]. Previous research has documented the presence of iron and manganese co-enrichment in plants such as rice, corn, and tomatoes [39,40]. In this study, the excessive accumulation of Fe and Mn probably contributed to the reduced plant root growth. This result was consistent with the study of Greipsson et al. [41].

TAC and MDA contents are used to indicate the redox state of plants [18] and levels of membrane damage [42]. Fig. 4c–d shows the changes in TAC and MDA in radish plants across all treatments. Treatment Cont-P-C showed a 23.84% decrease in TAC in shoots compared to plants grown in uncontaminated soil (NCont-P-C) because of Phe contamination. The addition of nZVI@BC or nZVI in Phe-polluted soil further decreased TAC to 0.97 ± 0.21 mmol Fe^2+^/g FW and 0.92 ± 0.07 mmol Fe^2+^/g FW, respectively. Conversely, the addition of nBC had no significant effect on TAC. The incorporation of nZVI resulted in higher MDA levels compared to nBC (Fig. 4d).

Oxidative stress was observed in this experiment after the addition of nZVI@BC. Despite a previous study demonstrating that both nZVI [43] and nBC [44] induced certain levels of plant oxidative damage, the observed oxidative damage did not increase as significantly as Fe and Mn contents in plants, especially in roots of NCont-P-Z. Furthermore, MDA contents in plants showed no significant differences. The stability of plants in challenging circumstances indicated that the significant increase of Fe and Mn in roots did not damage the plant interior to a certain extent. Furthermore, the comparison between Cont-P-Z and Cont-P-B treatments revealed that nZVI was the primary constituent of composite materials that induced oxidative stress and lipid peroxidation in roots, and the plant was more sensitive to nZVI.

### 3.5 Effects of nZVI@BC on soil bacterial community

**Fig. 5:**
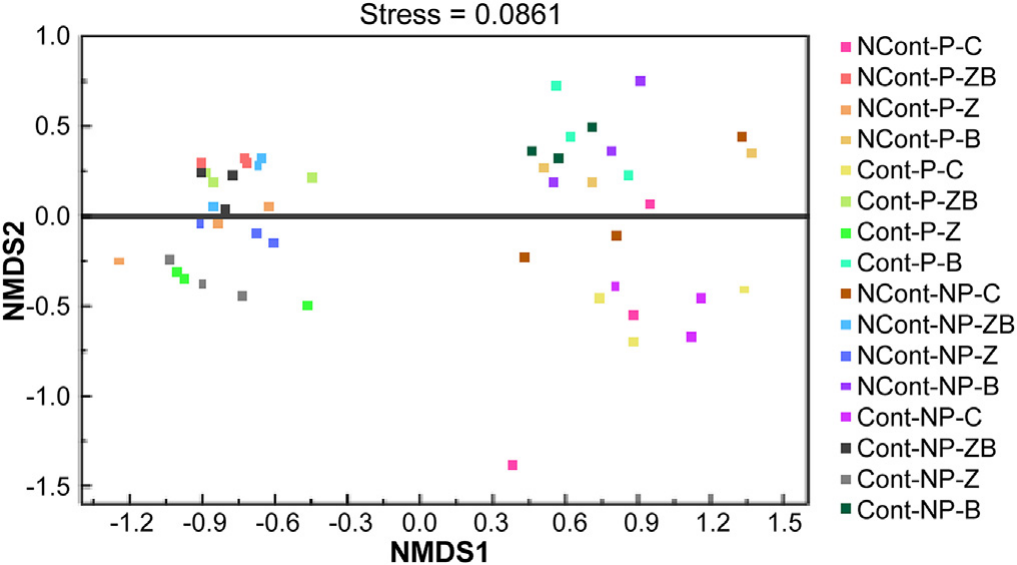
NMDS analysis of the bacterial communities in 16 treatments.

The bacterial community diversity was significantly influenced after the incorporation of nZVI@BC, nZVI, or nBC particles into the soil matrix (Text S6), suggesting that the addition of materials has profound long-term effects on soil microbes. Fig. 5 and Fig. S6 show that microbial communities were more sensitive to nZVI than nBC, which was consistent with the findings of Wu et al. [45] and Liu et al. [46]. Moreover, it was observed that radish roots in the treatments promoted the development of particular taxa in Phe-contaminated soil (Figs. S7 and S8), including *Flavisolibacter*, *Spingomonas*, *Massilia*, and *Lysobacter*, but depressed others, including *Bacillus*, *Micromonospora*, *Nocaedioides*, and *Streptomyces*. Furthermore, it was observed that the microbial response patterns in nZVI-treated soil were more similar to those observed with nZVI@BC than with nBC. The addition of nZVI and nBC differentially influenced the relative abundance of bacterial groups, such as *Bacteroidetes* and *β-proteobacteria*. However, their effects on *α-proteobacteria* were contrasting: nZVI treatments led to an increase in abundance, while nBC treatments caused a decrease. The impact of nZVI on *β-proteobacteria* was more pronounced than that of nBC, resulting in an overall increase of the *Proteobacteria* phylum. Similarly, it was observed that nZVI enriched *Acidobacteria* and depleted *Actinobacteria*, indicating particular variations in microbial populations.

## 4 Conclusion

The particles of nZVI@BC are promising composite nanomaterials for persulfate activation during soil remediation due to their low cost and environmental friendliness. However, the residue of nZVI@BC in soil was still effective in Phe degradation, transportation, and bioaccumulation. It also increased soil pH and DOC, affecting plant growth and microbial communities. In this study, the incorporation of nZVI@BC resulted in a 13.6% greater non-desorbed fraction of soil Phe and increased the total concentrations to 4.63 ± 0.21 mg/kg compared to Cont-P-C. The Fe content of radish shoots increased from 86.87 ± 5.61 mg/kg DW in NCont-P-C to 125.20 ± 11.93 mg/kg DW in NCont-P-ZB. The addition of nZVI@BC did not cause significant oxidative stress to plants. In addition, nZVI@BC could enrich bacteria related to the degradation of PAHs, such as *Lysobacter* and *Spingomonas*. In a comparison of the application effects of nBC and nZVI, nZVI@BC played a significant role in pollutant fixation and stabilized plant biomass, and nZVI@BC showed a better composite effect in soil-crop systems than the two materials added alone. To further enhance the practical application of these composites, future research should focus on optimizing the dosage of components like nZVI@BC. This optimization aims to strike an effective balance between food safety and remediation outcomes, ensuring that while environmental contaminants are efficiently managed, the safety and health of plant produce are not compromised.

## CRediT authorship contribution statement

**Lianzhou Shen:** Writing – original draft, Visualization, Methodology, Formal analysis, Data curation, Conceptualization. **Yue Cai:** Writing – review & editing, Methodology, Conceptualization. **Juan Gao:** Writing – review & editing, Formal analysis, Conceptualization.

## Declaration of competing interests

The authors declare that they have no known competing financial interests or personal relationships that could have appeared to influence the work reported in this paper.
