# Supplementary material for: Effects of nanoscale zero-valent iron loaded biochar on the fate of phenanthrene in soil-radish (Raphanus sativus L. var.radculus pers) system
Source: Eco Environ Health. 2025 Jan 22;4(1):100134. doi: 10.1016/j.eehl.2025.100134 (PMC11879671; doi:10.1016/j.eehl.2025.100134)
Supplement: Multimedia component 1 [file mmc1.docx]

**Supporting Information**

**Effects of nanoscale zero-valent iron loaded biochar on the fate of phenanthrene in soil-radish (*Raphanus sativus* L*. var.radculus pers*) system**

Lianzhou Shen ^a,b^, Yue Cai ^a,c^, Juan Gao ^a,b,^*

^a^ Institute of Soil Science, Chinese Academy of Sciences, Nanjing 211135, China

^b^ University of Chinese Academy Sciences, Nanjing college, Nanjing 211135, China

^c^ State Environmental Protection Key Laboratory of Environmental Health Risk Assessment, South China Institute of Environmental Science, Ministry of Ecology and Environment, Guangzhou 510655, China

*Corresponding author.

[juangao@issas.ac.cn](mailto:juangao@issas.ac.cn) (J. Gao)

**Text**

Text S1 Material Preparation.

Text S2 Measurements of antioxidant activity and elements.

Text S3 Extraction methods of different fractions of Phe.

Text S4 The instrument parameters of GC-MS.

Text S5 DNA extraction and Illumina MiSeq sequencing method.

Text S6 Responses of microbial community diversity to nZVI@BC.

**Figure**

Fig. S1 Diagram of experimental setup.

Fig. S2 DLS particle size analysis for nZVIBC.

Fig. S3 XPS spectra of overall survey for fresh and used nZVIBC.

Fig. S4 Photograph of radish seedlings in eight treatments after 5 d.

Fig. S5 Rarefaction Curve of the bacterial community diversity

Fig. S6 Venn diagram of the bacterial community in 8 treatments.

Fig. S7 Heatmaps of the bacterial community in 16 treatments.

Fig. S8 LEfSe analyze of the bacterial community in 16 treatments.

**Table**

Table S1 Overview of Experimental Group Designations

Table S2 Soil physicochemical properties in different treatments

Table S3 α-diversity of the bacterial community in different treatments.

**Text S1**

Material preparation

Two 25 L ball-mill tanks (DZ-25L, Tianchuang Powder Technology, Changsha, China) were used for preparation. Each tank was filled with 2 kg of steel balls with diameters of 8 mm, 10 mm, 12 mm, and 16 mm. Additionally, 6 kg of a 1:1 mixture of biochar and ZVI powder (w/w) was added to each tank. The program was set as follows: operating temperature, 25 °C at the speed of 120 rpm, a running time of 5 min, an intermittent time of 2 min, the reversing function on, and a total running time of 6 h. After starting and stopping, the lifting and discharging machine was used to move the tank to the vibrating screen discharging position and secure it. The ball mill cover was then replaced with the sieving discharge cover. After fixing the plug bolt and rotating the rotating arm to invert the ball-mill jar, the vibrating sieving switch was started for discharging, and the sieved material was collected as the prepared material. We followed the same procedure to prepare nZVI and nBC particles, except that only ZVI or biochar powder was used as the raw material. To prevent material mixing, clean ball mill beads of the same size and clean the ball mill jar were changed after each preparation.

**Text S2**

The instrument parameters of GC-MS

The analytical conditions for GC-MS utilized an SH-Rxi-5SiL MS chromatographic column (30 m × 0.25 mm × 0.25 μm film thickness) with an inlet temperature of 250 °C. Samples were injected in a non-split mode. The GC oven followed a program starting from 60 °C and ramping up to 260 °C at a rate of 10 °C/min. After a 1-min hold at 260 °C, the temperature was further increased to 300°C at a rate of 15 °C/min and maintained at this temperature for 3 min. The MS employed an electron bombardment ion source with selected ion monitoring (SIM). The ion source temperature was set at 200 °C, and the interface temperature was maintained at 250 °C.

**Text S3**

Extraction methods of Phe in plant, total and different fractions in soil

Six milliliter mixture solution of n-hexane and dichloromethane (1:1, v:v) was added as the extractant. The mixture was oscillated at 200 rpm for 2 h, ultra-sonicated in an ice-water bath for 30 min, and then centrifuged at 4000 rpm for 25 min. After three repeated steps, the extractant supernatants were added together and transferred into a solid-phase extraction column (SPE column). The columns were filled with a glass fiber sieve plate, 0.5 g anhydrous sodium sulfate, 1.0 g modified sulfonated silica gel, 1.0 g anhydrous sodium sulfate, and another glass fiber sieve plate from bottom to top, and pre-activated with 5 mL dichloromethane and 5 mL n-hexane before using. Subsequently, 15 mL of n-hexane and dichloromethane (1/1, v/v) were added to the column to elute the residual Phe and Pyr. All liquid passing through the column was collected and concentrated to less than 1 mL using a rotary evaporation at 40 °C, then reconstituted with dichloromethane to a final volume of 1 mL for analysis.

Extraction methods for soil Phe in different fractions

Desorbing fraction: 0.2 g soil sample was added into a 25 mL glass centrifuge tube, and 8 mL of a 50 mmol/L hydroxypropyl-β-cyclodextrin solution containing 0.05% NaN_3_ was added. The mixture was then shaken at 200 rpm for 12 h and subsequently centrifuged at 4000 rpm for 25 min. Five milliliters of the supernatant was transferred, and 3 mL of dichloromethane (DCM) was added. The resulting mixture was shaken at 200 rpm for 2 h. One milliliter of the upper organic phase was passed through a 0.22 μm organic filter membrane together with Na_2_SO_4_, which was then analyzed by a GC-MS.

Non-desorbing fraction: the soil sample remaining after the extraction of the desorbing fraction was washed with 15 mL of ultrapure water. It was vortexed for 10 seconds and centrifuged again for separation. After repeating these steps twice, the soil samples were lyophilized under a vacuum. Then, 5.0 mL of a mixed extractant (DCM:acetone = 1:1, v/v) was added, and the extraction was sonicated for 30 min. The organic phase was transferred to a tube after centrifugation at 4000 rpm for 25 min, and the extraction was repeated twice. The extractant was rotary evaporated till <1.0 mL, and then adjusted with DCM to 1.0 mL, and it was passed through Na_2_SO_4_ and a 0.22 μm organic filter membrane. Finally, it was analyzed with a GC-MS.

Bound residue: The soil sample remaining after the extraction of the non-desorbing fraction was air-dried. It was then treated with 10 mL of 2 mol/L H_2_O_2_ and heated in a water bath at 100 °C for 2 h. After cooling, the mixture was centrifuged at 4000 rpm for 25 min, and the supernatant was transferred into a glass tube. To this solution, 6 M HCl was added until the pH was less than 2.0. The resulting solution was further treated with 5 mL of DCM and sonicated for 30 min for liquid-liquid extraction. Two milliliters of the organic phase were rotary evaporated to dryness, adjusted to 1 mL with dichloromethane, passed through Na_2_SO_4_ and a 0.22 μm organic filter membrane, and then determined and analyzed by GC-MS.

**Text S4**

Measurements of antioxidant activity and elements

0.05 g of fresh plant samples were homogenized with 0.45 mL of physiological saline in an ice water bath. The mixture was then centrifuged, and the supernatant was separated and obtained as the original homogenate. A portion of the homogenate was pre-processed following Benzie's method, and its Ferric Reducing Ability of Plasma (FRAP) was measured using a microplate reader (Sunrise, Tecan, Austria). Malondialdehyde (MDA) content was determined using total protein (TP) and MDA kits from Nanjing Jiancheng Bioengineering Research Institute. Absorbance readings were taken at 562 nm and 532 nm with the microplate reader.

For each powder sample (0.05 g), it was mixed with 10 mL of digestion solution prepared using H_2_O_2_ and HNO_3_ in a 1:4 (v/v) ratio. After an overnight cold digestion, the mixtures were placed in a digestion oven. Initially, the oven was maintained at 170 ℃ until the solution became colorless and transparent, and then it was heated to 220°C till the solution almost evaporated. The remaining solution was diluted to a final volume of 10 mL with 3 mol/L HNO_3_, filtered using a 0.22 µm membrane, and analyzed using an inductively coupled plasma-atomic emission spectrometer (ICP-AES, Avio 200, Waltham Perkin Elmer, USA).

**Text S5**

DNA extraction and Illumina MiSeq sequencing method

DNA was extracted from rhizosphere soils using an OMEGA Soil DNA kit (M5635-02, Omega Bio-Tek, USA). Next, a pair of primers, 338F (ACTCCTACGGGAGGCAGCA) and 806R (GGACTACHVGGGTWTCTAAT), were used to amplify the V3-V4 regions of bacterial 16S rRNA using a thermocycler polymerase chain reaction (PCR) system (2720, ABI, USA). PCR amplicons were purified with VAHTSTM DNA Clean Beads (Vazyme, China) and quantified using the Quant-iT PicoGreen dsDNA Assay Kit (Invitrogen, USA). After the individual quantification step, amplicons were pooled in equal amounts, and pair-end 2 × 250 bp sequencing was performed using the Illumina MiSeq platform with MiSeq Reagent Kit v3 at Shanghai Personal Biotechnology Co., Ltd (China).

Microbiome bioinformatics and sequence data analyses were mainly performed with Quantitative Insights Into Microbial Ecology (QIIME, 2019.4). A reading of approximately 500 bp was obtained from each sample in Illumina sequencing, and the sequences were then quality filtered, denoised, merged and chimera removed using the DADA2 plugin. Non-singleton amplicon sequence variants (ASVs) were aligned with MAFFT and used to construct a phylogeny with fasttree2. Taxonomy was assigned to ASVs using the classify-sklearn naive Bayes taxonomy classifier in feature-classifier plugin against the SILVA Release 132 Database. Alpha-diversity indices of the bacterial community, including Chao1, Shannon index and Simpson index etc. were calculated using the ASV table in QIIME2. Beta-diversity analysis was performed to investigate the structural variation of microbial communities across samples using visualized via principal coordinate analysis (PCoA), nonmetric multidimensional scaling (NMDS). Venn diagram was generated to visualize the shared and unique ASVs among samples or groups using R package “VennDiagram”, based on the occurrence of ASVs across samples regardless of their relative abundance. Linear discriminant analysis effect size (LEfSe) was performed to detect differentially abundant taxa across groups using the default parameters.

**Text S6**

Responses of microbial community diversity to nZVI@BC

The structure of A comprehensive identification of all samples provided a total of 34,810 Amplicon Sequence Variants (ASVs), representing an extensive coverage exceeding 96.8%. Interestingly, the alpha diversity of Phe-contaminated soils was significantly affected, as indicated by the observed changes in the Chao1 and Shannon indices, particularly after nZVI treatment. The Chao1 index of NCont-P-ZB was found to be 4546 ± 285.9, which was significantly higher than that of NCont-N-C, but did not exceed the value of NCont-P-B (4818 ± 274.3). The observed Shannon index of 9.69 ± 0.26 for NCont-P-ZB suggested the presence of a varied bacterial population, although it was slightly lower than that of Cont-NP-C (10.27 ± 0.13). However, the Chao1 index for the Cont-NP-ZB treatment was observed as 4400 ± 238.8, but the Shannon index was determined to be 9.82 ± 0.04. These results indicate that the influence of nZVI@BC amendment on bacterial community was sufficient (Table S3). The visual representation of these effects can be observed in Fig. S5, which collectively depict the changes in composition and structure of the microbial community.


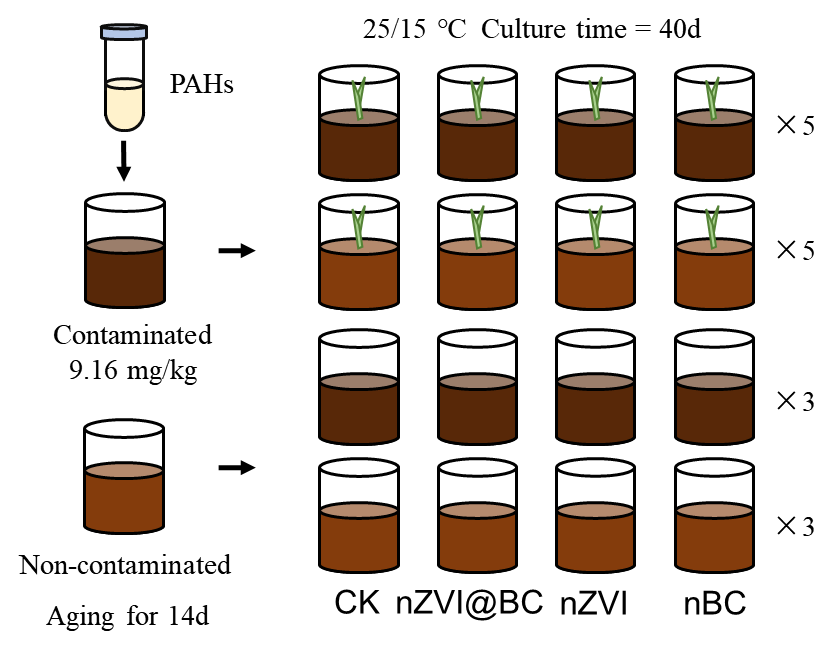


**Fig. S1** Diagram of experimental setup.

**Fig. S2** Size distribution of nZVIBC analyzed by DLS.

**Fig. S3** XPS spectra of fresh and used nZVIBC.


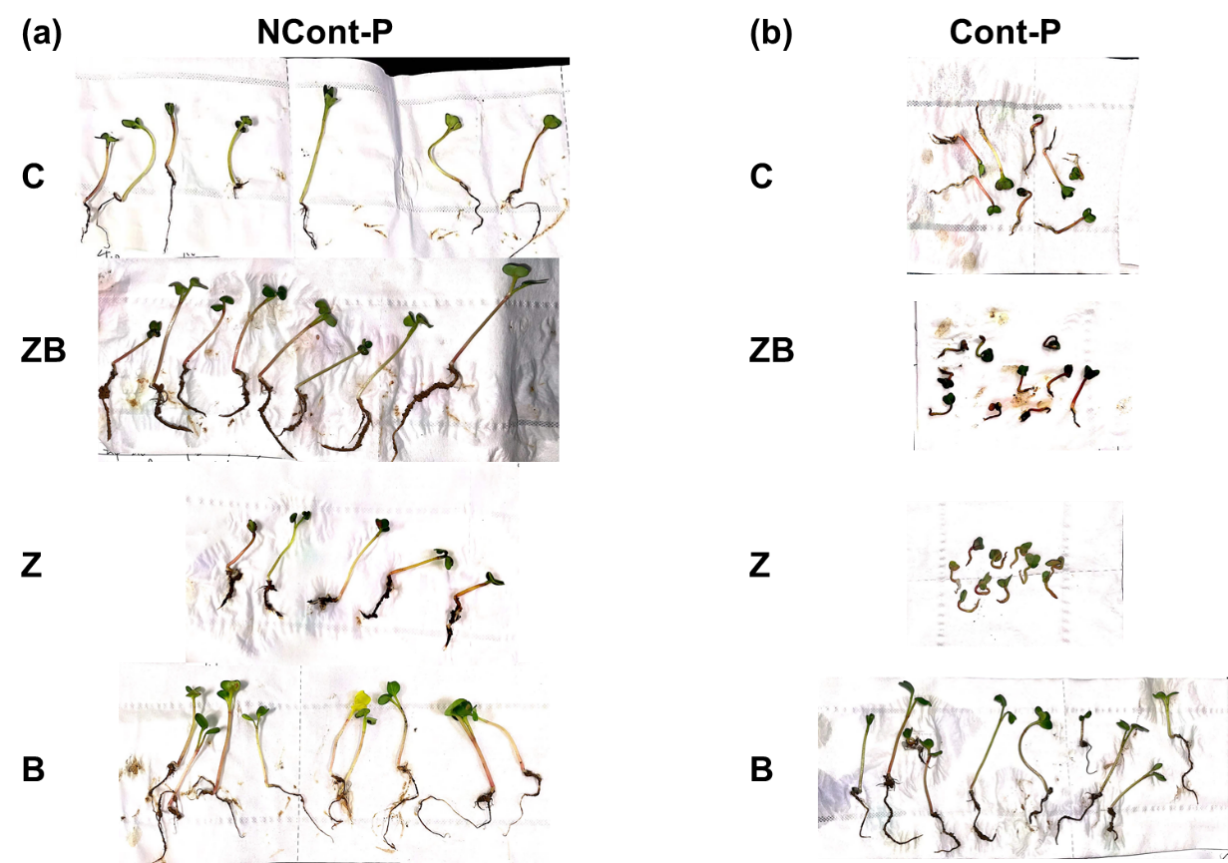


**Fig. S4** Photographs of radish seedlings in eight treatments after 5 d, a) in clean soil, and b) in Phe-polluted soil. Cont: Treatment involving soil that is contaminated. NCont: Treatment using soil that is not contaminated. P: Treatment where plants are introduced into the system. NP: Treatment without the introduction of plants. Z: Treatment with the addition of nZVI. B: Treatment involving the addition of nBC. ZB: Treatment that includes the combined addition of nZVI@BC. C: Treatment where no amendment materials are added, serving as a baseline condition.

**Fig. S5** Rarefaction Curve of the soil bacterial community diversity in 16 treatments.

**Fig. S6** Venn diagram of the soil bacterial community in 8 treatments, a) Comparison of treatments with and without Phe-contaminated or planted presence in no material addition condition. b) Comparison of Phe-contaminated and planted treatments with different materials addition.


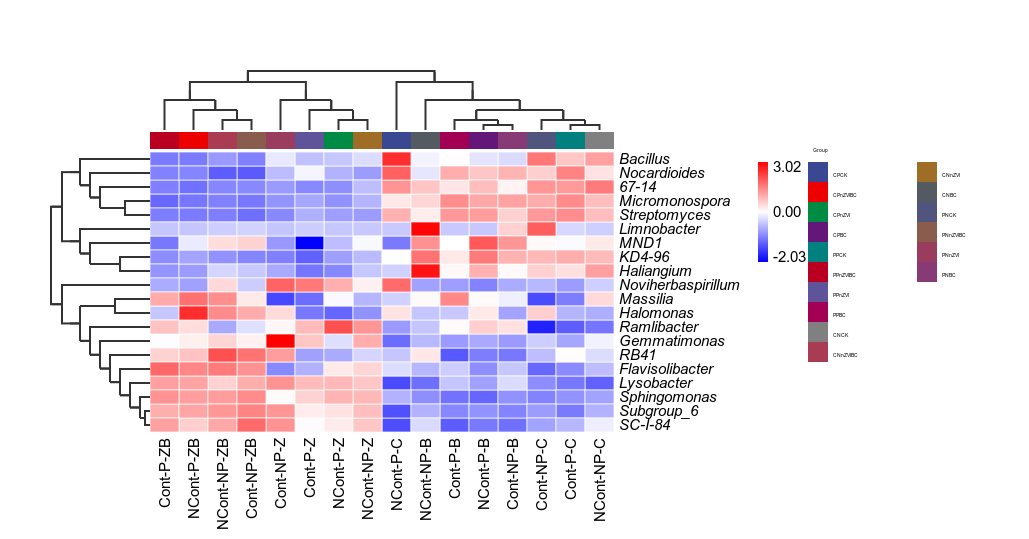


**Fig. S7** Heatmaps of the soil bacterial community in 16 treatments.

The samples were clustered UPGMA according to the euclidean distance of the species composition data, and arranged according to the clustering results. The horizontal group is microorganism, and the vertical group is treatment group. In the legend, red represents a positive correlation, blue represents a negative correlation, and the depth of the color corresponds to the correlation


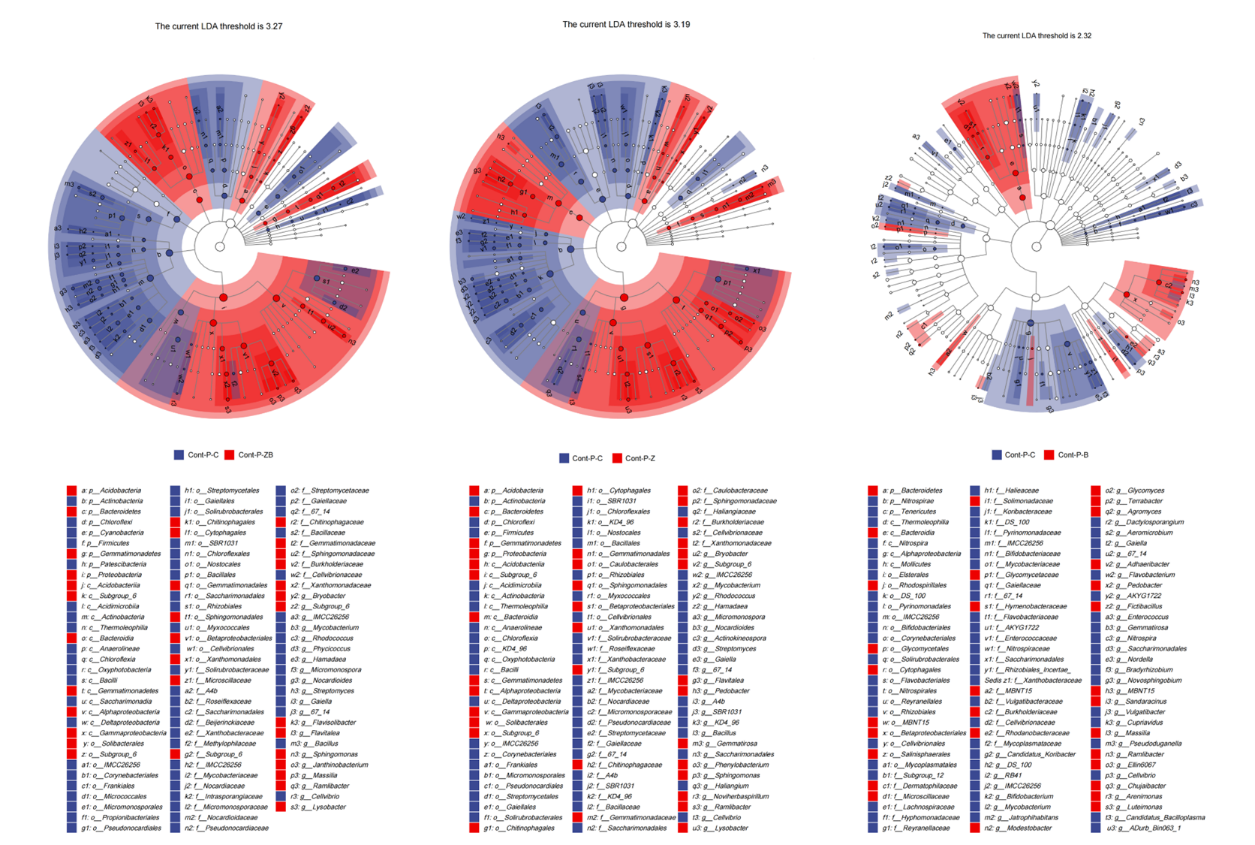


**Fig. S8** LEfSe analyze of the bacterial community in 16 treatments.

**Table S1** Overview of Experimental Group Designations

|  | **Contaminated (Cont)** | | **Non-contaminated (NCont)** | |
| --- | --- | --- | --- | --- |
|  | No Plant (NP) | Plant (P) | No Plant (NP) | Plant (P) |
| **No amendment (C)** | Cont-NP-C | Cont-P-C | NCont-NP-C | NCont-P-C |
| **nZVI@BC (ZB)** | Cont-NP-ZB | Cont-P-ZB | NCont-NP-ZB | NCont-P-ZB |
| **nZVI (Z)** | Cont-NP-Z | Cont-P-Z | NCont-NP-Z | NCont-P-Z |
| **nBC (B)** | Cont-NP-B | Cont-P-B | NCont-NP-B | Ncont-P-B |

Cont: Treatment involving soil that is contaminated. NCont: Treatment using soil that is not contaminated. P: Treatment where plants are introduced into the system. NP: Treatment without the introduction of plants. Z: Treatment with the addition of nZVI. B: Treatment involving the addition of nBC. ZB: Treatment that includes the combined addition of nZVI@BC. C: Treatment where no amendment materials are added, serving as a baseline condition.

**Table S2** Soil physicochemical properties in different treatments

| Treatment | Amendment | pH | DOC (mg/kg) |
| --- | --- | --- | --- |
| NCont-NP | C | 6.91 ± 0.19 | 116 ± 28 |
|  | ZB | 7.06 ± 0.02 | 131 ± 12 |
|  | Z | 7.00 ± 0.06 | 112 ± 4 |
|  | B | 7.07 ± 0.03 | 151 ± 24 |
| NCont-P | C | 6.92 ± 0.02 | 111 ± 7 |
|  | ZB | 7.02 ± 0.04 | 115 ± 3 |
|  | Z | 6.99 ± 0.06 | 101 ± 4 |
|  | B | 7.15 ± 0.03 | 128 ± 13 |
| Cont-NP | C | 6.82 ± 0.05 | 82.4 ± 2.5 |
|  | ZB | 6.90 ± 0.02 | 88.7 ± 5.6 |
|  | Z | 6.93 ± 0.03 | 74.1 ± 5.4 |
|  | B | 6.98 ± 0.04 | 112 ± 11 |
| Cont-P | C | 6.69 ± 0.05 | 79.0 ± 4.10 |
|  | ZB | 6.81 ± 0.07 | 86.0 ± 6.0 |
|  | Z | 6.78 ± 0.05 | 80.9 ± 4.4 |
|  | B | 6.91 ± 0.15 | 118 ± 25 |

**Table S3** α-diversity of the bacterial community in different treatments

| Treatments | Material | Chao1 | Shannon | Simpson |
| --- | --- | --- | --- | --- |
| NCont-NP | C | 4159 ± 342^b^ | 9.91 ± 0.40^abcdef^ | 0.994 ± 0.003 |
|  | ZB | 4441 ± 235^ab^ | 9.78 ± 0.15^cdef^ | 0.992 ± 0.002 |
|  | Z | 4295 ± 30^ab^ | 9.70 ± 0.06^def^ | 0.991 ± 0.003 |
|  | B | 4604 ± 402^ab^ | 10.2 ± 0.3^abc^ | 0.996 ± 0.001 |
| NCont-P | C | 4775 ± 63^a^ | 10.3 ± 0.1^a^ | 0.996 ± 0.001 |
|  | ZB | 4546 ± 286^ab^ | 9.69 ± 0.26^def^ | 0.991 ± 0.003 |
|  | Z | 4090 ± 99^b^ | 9.67 ± 0.22^ef^ | 0.994 ± 0.002 |
|  | B | 4818 ± 274^a^ | 10.1 ± 0.2^abcd^ | 0.995 ± 0.002 |
| Cont-NP | C | 4760 ± 536^a^ | 10.3 ± 0.1^ab^ | 0.996 ± 0.001 |
|  | ZB | 4400 ± 239^ab^ | 9.82 ± 0.04 ^bcdef^ | 0.993 ± 0.001 |
|  | Z | 4423 ± 140^ab^ | 9.69 ± 0.14^def^ | 0.991 ± 0.001 |
|  | B | 4562 ± 346^ab^ | 10.0 ± 0.4^abcde^ | 0.992 ± 0.006 |
| Cont-P | C | 4836 ± 15^a^ | 10.2 ± 0.1^ab^ | 0.995 ± 0.002 |
|  | ZB | 4576 ± 260^ab^ | 9.78 ± 0.1^cdef^ | 0.992 ± 0.002 |
|  | Z | 4127 ± 255^b^ | 9.47 ± 0.21^f^ | 0.991 ± 0.003 |
|  | B | 4880 ± 207^a^ | 10.1 ± 0.2^abcd^ | 0.995 ± 0.001 |

Correlation analysis was carried out according to the mean value of the group. Chao1, Shannon, and Simpson represent richness, diversity, and evenness, respectively
